# Impact of HDV infection on post-transplant outcomes in patients transplanted for HBV-related liver disease: results from a multicenter cohort study in Southern Italy

**DOI:** 10.1007/s15010-025-02707-5

**Published:** 2025-12-15

**Authors:** Gianfranca Stornaiuolo, Mariantonietta Pisaturo, Lorenzo Salmoni, Antonio Russo, Debora Angrisani, Giovanni Valente, Francesco Longobardi, Antonella Santonicola, Filomena Morisco, Maria Stanzione, Alfonso Galeota Lanza, Rosaria Focareta, Caterina Sagnelli, Carmine Coppola, Nicola Coppola

**Affiliations:** 1 Infectious Diseases Unit, AOU Vanvitelli, Naples, Italy; 2 Department of Mental and Physical Health and Preventive Medicine, University of Campania “Luigi Vanvitelli”, Naples, Italy; 3 Department of Life Science, Health, and Health Professions Link Campus University, Via del Casale di San Pio V, 44, 00165 Rome, Italy; 4 Hepatology Unit, AORN Cardarelli, Naples, Italy; 5 Hepatic Pathophysiology SATTE, AORN Sant’Anna E San Sebastiano, Caserta, Italy; 6 Internal Medicine and Hepatology Unit, Gragnano Hospital, Naples, Italy; 7 Gastrointestinal Unit, Department of Medicine Surgery and Dentistry, University of Salerno, Salerno, Italy; 8 Liver and Biliary Diseases Unit, University of Naples Federico II, Naples, Italy; 9 Department of Mental Health and Public Medicine, Section of Infectious Diseases, University of Campania Luigi Vanvitelli, Via L. Armanni 5, 80131 Naples, Italy

**Keywords:** Liver transplantation, HBV infection, HDV infection, Clinical events

## Abstract

**Aims:**

Patients with HBV and HBV/HDV infection were compared in terms of clinical outcomes after liver transplantation in a long-term follow-up.

**Methods:**

In a multicenter retrospective study, the patients, who had received liver transplants for HBV chronic liver disease, were enrolled in a long-term follow-up (1–20 years). The primary outcome was overall survival, the secondary outcome occurrence of clinical events.

**Results:**

A total of 257 patients were enrolled, 95 with HBV (HBV group) and 162 with HBV/HDV (HBV/HDV group) infection. Overall, 31 patients died in the follow-up, most frequently due to extrahepatic events. Overall mortality was similar between the two groups (15.8% in HBV vs 9.9% in HBV/HDV group), while deaths from hepatic events were more frequent in the first group (7.4 vs. 1.2%, p = 0.014). In multivariable logistic regression analysis only a history of chronic kidney disease (CKD) at the time of transplantation emerged as independent factor associated with death (OR 7.027, 95% CI 2.068–23.876; p = 0.002).

Overall, 84 patients experienced at least one clinical event, mainly onset of renal failure (57 cases), cancer (32) and hepatic clinical (19). Compared with HBV/HDV group, clinical events occurred more frequently in HBV group (44.2 vs. 25.9%; p = 0.003), both hepatic and extrahepatic (11.6 vs. 4.9%, p = 0.050; 35.8 vs. 23.5%, p = 0.034, respectively). In multivariable logistic regression analysis, HBV group (OR 2.243, 95% CI 1.172–4.293; p = 0.015) and CKD at the time of transplantation were associated to the occurrence of clinical events (OR 8.890, 95% CI 2.373–33.306; p = 0.001).

**Conclusions:**

In this long-term follow-up study, HDV infection was not associated to a worsen post-transplant outcomes, while chronic kidney disease at transplantation emerged as the strongest predictor of mortality and clinical events.

**Supplementary Information:**

The online version contains supplementary material available at 10.1007/s15010-025-02707-5.

## Introduction

The World Health Organization (WHO) estimated that 254 million people in 2022 were living with chronic hepatitis B virus (HBV) infection, with around 1.2 million new infections annually [1]. In the same year, WHO reported that about 1.3 million people died from viral hepatitis, with HBV accounting for 83% of these deaths [1]. Approximately one-third of people with chronic HBV infection develop long-term complication, such as cirrhosis, end-stage liver disease, or hepatocellular carcinoma (HCC) [2]; in these conditions, liver transplantation represents a key therapeutic tool.

One of the most relevant factors associated with a severe prognosis in chronic HBV subject is the coinfection with hepatitis D virus (HDV). The prevalence of HDV infection among hepatitis B surface antigen (HBsAg) positive population is estimated to range between 13.02 and 16.4% [3–7]. Recently, Polaris Observatory re-evaluated global data and found a lower HDV prevalence in most of 25 countries analyzed (18/25) compared to previous data. In Italy, for example, the weighted average prevalence of anti-HDV positivity was estimated at 3.4% [6].

HBV/HDV chronic co-infections are associated with more severe liver disease, leading to a high risk of developing cirrhosis within 5 years and HCC within 10 years [5, 8], compared to HBV mono-infected patients.

Before the availability of specific antiviral therapy, liver transplantation was often considered the last and only option for survival [9, 10]. However, a favorable prognosis and good long-term outcomes were usually shown in HBV/HDV transplanted patients [11–13].

The knowledge about HDV infection has improved, and new specific treatments are now available. Currently, in the evaluation of natural history of chronic HDV infection, HDV RNA positivity is recognized as the main negative prognostic factor: viremic patients have an approximately 3.8-fold higher risk of developing hepatic complications compared with anti-HDV–positive but non-viremic individuals [5, 14, 15]. Moreover, long-term use of bulevirtide seems to be associated with sustained control of both infection and disease in at least half of patients [5, 14, 15]. Finally, evidence suggesting a better prognosis for HBV/HDV-transplanted patients compared with those with HBV alone derives from older studies, performed when strategies for HBV control after liver transplantation were limited.

The present study aimed to evaluate the impact of HDV infection on the long-term clinical outcomes in patients with HBV infection who underwent liver transplantation.

## Methods

### Study design and setting

A retrospective observational multicenter study was conducted across six liver units in the Campania, region of southern Italy. These centers serve as specialized referral hubs for the management of patients in the pre- and post-liver transplantation phases. They have jointly participated in numerous clinical investigations, using standardized clinical and laboratory protocols. Shared management procedures for both perioperative and post-transplant care have been established, promoting uniformity in clinical practice. Furthermore, regular multidisciplinary meetings are held to discuss and manage the complex cases. This integrated and harmonized approach ensures consistency in post-transplant care and facilitates continuous exchange of clinical expertise across centers.

Eligible participants were adult patients who underwent liver transplantation between 1991 and 2024 due to complications arising from either HBV infection alone (HBV group) or HBV and HDV coinfection (HBV/HDV group), with a minimum follow-up of one year at one of the six participating centers. Since HDV RNA test has not been available in all the centers participating to the study until 2016, in the present study anti-HDV status at transplantation was used to define the two groups of patients.

Patients were excluded if HDV antibody status was unavailable at the time of transplantation.

An electronic centralized database was used using data extracted from patient medical records. Serological markers (HBsAg, HBV-DNA, anti-HDV) were collected in the 30 days preceding the transplantation. Variable collected included hematological and biochemical parameters, viral hepatitis serological markers, antiviral and immunosuppressive treatments, comorbidities, virological status before transplantation, and post-transplant clinical events.

### Outcome measures

The primary outcome of the study was overall survival, and the secondary outcome the occurrence of clinical events.

Liver-related clinical events included ascites, variceal bleeding, hepatic encephalopathy, acute or chronic rejection, serious infections, recurrence of hepatocellular carcinoma, cirrhosis and HBV relapse (defined as retro conversion to HBsAg positivity and/or HBV DNA detectability in serum).

Extra-hepatic clinical events included onset of renal failure, cancer, diabetes and cardiovascular complications.

Both primary and secondary outcomes were analyzed according to the presence or not of HDV infection.

The study also investigated the clinical, virological, and demographic characteristics associated with the overall survival and the development of clinical events. Because of the retrospective and multicenter nature of the study, the completeness of clinical and laboratory variables varied across centers. Therefore, comparative analyses were restricted to parameters with high data availability, and an available-case approach was adopted for each variable. Parameters with excessive missingness were excluded from inferential analyses to reduce bias.

### Statistical analysis

For the descriptive analysis, categorical variables were presented as absolute numbers and their relative frequencies. Continuous variables were summarized as the median and interquartile range (Q1–Q3). We performed a comparison between HBV and HBV/HDV patients and sub-analysis including clinical outcomes and mortality with Pearson chi-square or Fisher’s exact test for categorical variables and Student’s t-tests or Mann–Whitney tests for continuous variables. To evaluate the distribution of continuous variables, we used the Shapiro–Wilk test. Odds ratios were calculated using binomial logistic regression; these analyses were performed including clinically relevant parameters or statistically significant parameters through a univariate analysis. A *p*-value below 0.05 was considered statistically significant. Analyses were performed by STATA version 16.

### Ethical considerations

The study was approved by the Ethics Committee of the University of Campania L. Vanvitelli, Naples (n°24,936/2024). All procedures performed in this study were in accordance with the ethics standards of the institutional and/or national research committee and with the 1964 Helsinki declaration and its later amendments or comparable ethics standards. For deceased patients, data processing was carried out under Article 110-bis of Regulation (EU) 2016/679 (GDPR). Informed consent was obtained from all participants included in the study.

## Results

We enrolled 257 patients who underwent liver transplantation due to complications related to chronic HBV infection. Baseline demographic and clinical characteristics of the patients at transplantation are summarized in Supplementary Table 1.

### Characteristics of enrolled patients at the time of liver transplantation

Among the 257 enrolled patients, 95 (36.96%) had HBV monoinfection (HBV group) and 162 (63.04%) had HBV/HDV coinfection (HBV/HDV group).

Table 1 showed epidemiological and clinical characteristics of patients in the two groups of patients at the time of the transplantation with demographic characteristics comparable (Table 1). Although most of the patients in both groups were transplanted for cirrhosis, the frequency of cirrhosis in HBV/HDV group was higher than in HBV group (100 vs 94.6%, p = 0.020). Conversely, HCC at transplantation was more common in the HBV group, though not significantly (63 vs 58%, p = 0.490) (Table 1). At the time of liver transplantation, 7 patients (7.4%) in HBV group and 19 patients (12%) in HBV/HDV group were HCV coinfected (Table 1). HDV-RNA was available in only 18 (11.1%) of the 162 anti-HDV–positive patients, 17/18 (94.4%) were viremic.

**Table 1:** demographic and clinical data at the time of liver transplantation according to the presence or not of HDV infection

	HBV group	HBV/HDV group	p
N° of patients	95 (36.96%)	162 (63.04%)	
Gender M n° (%)	75 (78.9%)	119 (73.5%)	0.323
Age at transplant, Median and IQR	56 (48; 62)	55 (50; 61)	0.975
No° (%) patients with			
- IVDA	7 (7.5%)	14 (8.8%)	0.723
- Ethyl abuse	19 (20.2%)	19 (12.0%)	0.079
- Familiarity with HbsAg	28 (32.6%)	48 (37.8%)	0.434
Years of follow-up Median (IQR)	6 (4; 15)	7 (3; 11)	0.897
No° (%) transplant patients			0.064
- < 2010	31 (32.6%)	34 (21.1%)	
- 2010–2015	15 (15.8%)	46 (28.6%)	
- 2016–2020	34 (35.8%)	55 (34.2%)	
- > 2020	15 (15.8%)	26 (16.1%)	
Transplanted for			
- Cirrhosis n (%)	74 (94.9%)	127 (100%)	0.020
- HCC n (%)	46 (63.0%)	69 (58.0%)	0.490
No° (%)/ tested transplant patients			
- HBV DNA neg	47/64 (73.4%)	56/68 (82.4%)	0.216
- Anti-HCV	7/94 (7.4%)	19/158 (12.0%)	0.248
- Anti-HIV	0/90 (0%)	0/98 (0%%)	–
No° (%)/ tested transplant patients			
- With diabetes mellitus	14 (18.9%)	27 (24.8%)	0.351
- CKD	5 (6.6%)	11 (10.4%)	0.372
- Heart disease	13 (17.1%)	12 (10.7%)	0.205

Missing data were excluded from this analysis; denominators reflect available cases

No significant differences were found between two groups regarding diabetes, chronic kidney and cardiac disease (Table 1).

During post-transplant follow-up, all patients were treated with nucleos(t)ide analogue.

### Mortality in follow-up

Overall, 31 patients died in the follow-up; however overall mortality and cause-specific mortality were similar between groups (Table 2), with extrahepatic events representing the most common cause of death.

**Table 2:** Mortality and occurrence of clinical events in post-transplant follow-up in enrolled patients, by HDV status

	HBV group	HBV/HDV group	p
N° (%) patients with			
- death related to hepatic event	7 (7.4%)	2 (1.2%)	0.014
- death not related to hepatic event	8 (8.4%)	14 (8.6%)	1.000
- overall deaths	15 (15.8%)	16 (9.9%)	0.170
N° (%) patients with liver-related death			0.417
- In the first 5 years after transplant	6 (85.7%)	1 (50.0%)	
- From the 6th to the 10th year post transplant	1 (14.3%)	1 (50.0%)	
After the 10th year post transplant	0 (0%)	0 (0%)	
N° (%) patients with death not related to hepatic events			0.523
- In the first 5 years after transplant	5 (62.5%)	8 (57.1%)	
- From the 6th to the 10th year post transplant	3 (37.5%)	4 (28.6%)	
After the 10th year post transplant	0 (0.0%)	2 (14.3%)	
N° (%)patients with overall death			0.235
- In the first 5 years after transplant	12 (80.0%)	9 (56.3%)	
- From the 6th to the 10th year post transplant	3 (20.0%)	5 (31.3%)	
After the 10th year post transplant	0 (0.0%)	2 (12.5%)	
N° (%) patients with at least one clinical event (hepatic or extra-hepatic)	42 (44.2%)	42 (25.9%)	0.003
N° (%)patients with at least one clinical event			0.521
- In the first 5 years after transplant	40 (95.2%)	37 (90.2%)	
- From the 6th to the 10th year post transplant	2 (4.8%)	3 (7.3%)	
- After the 10th year post transplant	0 (0.0%)	1 (2.4%)	
N° (%)patients with at least one hepatic event (cirrhosis, HCC, HBV relapse, decompensation)	11 (11.6%)	8 (4.9%)	0.050
N° (%)patients with at least one hepatic event			1.000
- In the first 5 years after transplant	10 (90.9%)	7 (87.5%)	
- From the 6th to the 10th year post transplant	1 (9.1%)	1 (12.5%)	
- After the 10th year post transplant	0 (0%)	0 (0%)	
N° (%) patients with at least one extra-hepatic event (renal failure, new onset neoplasm)	34 (35.8%)	38 (23.5%)	0.034
N° (%) patients with at least one extra-hepatic event			0.633
- In the first 5 years after transplant	32 (94.1%)	35 (92.1%)	
- From the 6th to the 10th year post transplant	2 (5.9%)	2 (5.3%)	
- After the 10th year post transplant	0 (0.0%)	1 (2.6%)	
N° (%) patients with			
- HBV relapse	0 (0.0%)	0 (0.0%)	–
- HCC	10 (10.5%)	3 (1.8%)	0.163
- Cirrhosis	3 (3.15%)	3 (1.8%)	0.003
- Hepatic decompensation	0 (0%)	2 (1.2%)	0.532
- Renal failure	27 (28.4%)	30 (8.2%)	0.065
- Onset of cancer	15 (15.7%)	17 (10.4%)	0.214

Missing data were excluded from this analysis; denominators reflect available cases

Deaths due to hepatic events were significantly more frequent in HBV group (7.4 vs. 1.2%, p = 0.014), while extrahepatic mortality was comparable (8.4 vs. 8.6%) (Table 2). In both groups, most deaths occurred within the first 5 years without statistically significant differences (p = 0.235) (Table 2).

The Table 3 showed the characteristics of the 31 patients died compared to 226 patients survived during the follow-up. The survivors were less frequently males (72.2 vs. 96.8%, P = 0.002) but had a similar median age at the transplant (57 years, IQR 46–63, vs. 55, IQR 49–61; p = 0.718) (Table 3). No significant differences were found considering the type of immunosuppressive regimen, antiviral treatment, or the presence of HDV infection (Table 3). However, chronic kidney disease (CKD) at transplantation was more frequently observed in patients who died (25 vs. 6.3%; P = 0.003) (Table 3). Interestingly, survivors were more often treated with subcutaneous anti-HBs immunoglobulins (43.8 vs. 28.6%, respectively; P = 0.015). (Table 3).

**Table 3:** demographic, virological and clinical data associated with overall mortality

	Non survivors	Survivors	p
N° of patients	31	226	
Gender M n° (%)	30 (96.8%)	164 (72.2%)	0.002
Age at transplant, Median and IQR	57 (46;63)	55 (49;61)	0.718
Median years of follow-up (IQR)	4 (2;9)	7 (4;12)	0.006
No. (%) of transplanted patients			0.769
- < 2010	9 (29.0%)	56 (24.9%)	
- 2010–2015	8 (25.8%)	53 (23.6%)	
- 2016–2020	11 (35.5%)	78 (34.7%)	
- > 2020	3 (9.7%)	38 (16.9%)	
Transplanted for			
- Cirrhosis n (%)	27 (100%)	174 (97.8%)	1.000
- HCC n (%)	15 (68.2%)	100 (58.8%)	0.399
Coinfection, n° (%)			
- HDV	16 (51.6%)	146 (64.6%)	0.160
- HCV	6 (20.7%)	20 (9.0%)	0.051
Comorbidities at the time of transplant no° (%)			
- Diabetes mellitus	8 (34.8%)	33 (20.6%)	0.128
- CKD	6 (25.0%)	10 (6.3%)	0.003
- Heart disease	1 (4.2%)	24 (14.6%)	0.210
At least 1 year history of immunosuppressive medication, no° (%)			0.554
- Cyclosporine	1 (3.4%)	19 (8.7%)	
- Tacrolimus	10 (34.5%)	68 (31.2%)	
- Mycophenolate	2 (6.9%)	4 (1.8%)	
- Everolimus	3 (10.3%)	25 (11.5%)	
- Sirolimus	0 (0.0%)	2 (0.9%)	
- Association therapies	13 (44.8%)	100 (45.9%)	
History of at least 1 year of anti-HBV therapy with NUC, n° (%)			0.381
- Lamivudine	4 (16.0%)	46 (23.2%)	
- Entecavir	15 (60.0%)	122 (61.6%)	
- Tenofovir	5 (20.0%)	28 (14.1%)	
- Telbivudine	0 (0.0%)	1 (0.5%)	
- Association therapies	1(4.0%)	1 (0.5%)	
History of at least 1 year of administration of anti-HBs immunoglobulin, n° (%)			
- Intravenous	7 (25.0%)	20 (9.5%)	0.125
- Intramuscular	13 (46.4%)	98 (46.7%)	0.981
- Subcutaneous	8 (28.6%)	92 (43.8%)	0.015

Missing data were excluded from this analysis; denominators reflect available cases

The multivariable logistic regression analysis evaluating predictors of mortality during follow-up showed CKD at the time of transplant emerged as the only independent clinical factor significantly associated with an increased risk of death (OR 7.027, 95% CI 2.068–23.876; p = 0.002) (Table 4).

**Table 4:** Multivariable logistic regression assessing condition associated to death during follow up

Variable included	OR	95% CI	p
Age at transplantation	1.035	0.981–1.091	0.213
Presence of CKD at transplantation	7.027	2.068–23.876	0.002
Performed subcutaneous immunoglobulins for HBV	0.555	0.212–1.456	0.232
HBV mono-infection	1.741	0.679–4.466	0.248

Gender not included considering the high rate of males in non-survivor group

### Occurrence of clinical events

Overall, 84 patients experienced at least one clinical event. The most frequent clinical events after transplantation were the renal failure (57 patients), and new-onset cancer, in 32 patients. Hepatic clinical complications occurred in only 19 patients, primarily new onsets of cirrhosis or HCC. No patient experienced during follow-up an HBV relapse.

The data on the occurrence of clinical events during the long-term follow-up according to the presence or not of HDV infection were shown in Table 2. Clinical events occurred more frequently in HBV group than in the HBV/HDV group (44.2 vs. 25.9%; p = 0.003). Instead, the timing of clinical event was similar in the groups (p = 0.521) (Table 2).

Both hepatic and extra-hepatic events occurred more frequently in HBV group (Table 2). Precisely, hepatic events occurred in 11.6% of patients in HBV group and in 4.9% of patients in HBV/HDV group (p = 0.050), extra-hepatic events in 35.8 and 23.5%, respectively (p = 0.034).

Table 5 showed the characteristics of the 84 patients that had clinical events versus the 173 without. When comparing patients with and without clinical events, no significant differences emerged in baseline demographic and clinical characteristics at the transplant (Table 5). However, those who experienced clinical events during follow-up were more likely to have HBV monoinfection (69.4 vs. 50.0%; P = 0.003) and CKD at transplant (17.6 vs. 2.8%; p = 0.001) compared to those without such events. (Table 5).

**Table 5:** demographic, virological and clinical data associated with at least one post-transplant event

	Patients with at least a clinical event	Patients without clinical event	p
No. of patients	84	173	
Gender male, n° (%)	65 (77.4%)	129 (74.6%)	0.623
Age at transplant, Median and IQR	57 (49; 62)	55 (49; 61)	0.297
Median years of follow-up (IQR)	6 (4; 12)	7 (4; 12)	0.743
No. (%) of transplanted patients			0.633
- < 2010	21 (25.3%)	44 (25.4%)	
- 2010–2015	20 (24.1%)	41 (23.7%)	
- 2016–2020	32 (38.6%)	57 (32.9%)	
- > 2020	10 (12.0%)	31 (17.9%)	
Transplanted for			
- Cirrhosis n (%)	75 (98.7%)	126 (97.7%)	1.000
- HCC n (%)	46 (63.9%)	69 (57.5%)	0.382
Coinfection, n° (%)			
- HDV	42 (50.0%)	120 (69.4%)	0.003
- HCV	9 (11.0%)	17 (10.0%)	0.811
Comorbidities at the time of transplant no° (%)			
- diabetes mellitus	19 (26.4%)	22 (19.8%)	0.298
- CKD	13 (17.6%)	3 (2.8%)	0.001
- Heart disease	11 (14.5%)	14 (12.5%)	0.696
At least 1 year history of immunosuppressive medication, no° (%)			0.182
- Cyclosporine	7 (8.6%)	13 (7.8%)	
- Tacrolimus	21 (25.9%)	57 (34.3%)	
- Mycophenolate	3 (3.7%)	3 (1.8%)	
- Everolimus	7 (8.6%)	21 (12.7%)	
- Sirolimus	2 (2.5%)	0 (0.0%)	
- Association therapies	41 (50.6%)	72 (43.4%)	
History of at least 1 year of anti-HBV therapy with NUC, n° (%)			0.847
- Lamivudine	17 (22.7%)	33 (22.3%)	
- Entecavir	44 (58.7%)	93 (62.8%)	
- Tenofovir	13 (17.3%)	20 (13.5%)	
- Telbivudine	0 (0.0%)	1 (0.7%)	
- Association therapies	1 (1.3%)	1 (0.7%)	
History of at least 1 year of administration of anti-HBs immunoglobulin, n° (%)			0.157
- Intravenous	13 (16.5%)	14 (8.8%)	
- Intramuscular	32 (40.5%)	79 (49.7%)	
- Subcutaneous	34 (43.0%)	66 (41.5%)	

Missing data were excluded from this analysis; denominators reflect available cases

The multivariable logistic regression analysis assessing predictors of clinically relevant post-transplant events showed that the presence of HBV mono-infection (OR 2.243, 95% CI 1.172–4.293; p = 0.015) was independently associated with the occurrence of clinical events (Table 6). However, the strongest predictor was the presence of chronic kidney disease (CKD) at the time of transplantation, which conferred a nearly ninefold increased risk (OR 8.890, 95% CI 2.373–33.306; p = 0.001) (Table 6).

**Table 6:** Multivariable logistic regression assessing the condition associated to a clinical relevant event

Variable included	OR	95% CI	p
Gender male	1.394	0.663–2.931	0.381
Age at transplant	1.025	0.989–1.062	0.179
HBV mono-infection	2.243	1.172–4.293	0.015
Presence of CKD at transplant	8.890	2.373–33.306	0.001

## Discussions

Our multicenter study analyzed a cohort of 257 patients who underwent liver transplantation for complications related to HBV infection or HBV/HDV co-infection, in a long-term follow-up up to 20 years. During the follow-up, 12.1% of the patients died and 32.7% experienced at least one clinical event, either hepatic or extrahepatic. Our findings on overall survival were consistent with the national data recently reported [16]. However, the present study provides complementary and region-specific information. First, Campania region is an area with a high prevalence of HBV/HDV coinfection that explained a high representation of HDV patients in the present study. Additionally, unlike registry-based reports, our cohort includes clinical and virological data, enabling an in-depth evaluation of extra-hepatic complications, comorbidities, and predictors of post-transplant events.

Interestingly, the HBV monoinfected group showed a significantly higher liver-related mortality (7.4 vs. 1.2%; p = 0.034). However, the only factor independently associated with overall mortality was the presence of pre-existing CKD at the time of transplantation. Clinical events occurred more frequently in the HBV monoinfected group compared to the HBV/HDV group (44.2 vs. 25.9%; p = 0.003). Multivariable logistic regression analysis further identified HBV mono-infection as an independent predictor of clinically relevant post-transplant events (OR 2.243, 95% CI 1.172–4.293; p = 0.015), highlighting that patients with HBV mono-infection have more than double the risk of developing complications after transplantation compared to those with HBV/HDV co-infection. The association between HBV monoinfection and higher rate of clinical events may be due the fact that HDV was known to act as a ‘dominant’ virus in HBV/HDV coinfection, suppressing HBV replication and reducing HBV-related necroinflammatory activity [17]; this virological dominance may lead to a less aggressive HBV-driven clinical course after transplantation.

The recurrence rate of hepatocellular carcinoma observed in our cohort was unexpectedly low (1.8% during a median follow-up of 7 years). Several factors may contribute to this finding: the pre-transplant downstaging and bridging protocols used may have reduced the recurrence risk after transplantation; a close post-transplant surveillance protocol after transplantation may potentially have enabled early detection and management of neoplastic progression; the widespread use of high-barrier nucleos(t)ide analogues may have reduced the risk of de novo HCC in HBV-related disease. However, given the retrospective and multicenter nature of the cohort, the possibility of underdiagnosis or incomplete documentation cannot be fully excluded, and this factor should be considered when interpreting the recurrence rate.

Our data suggested that, in the context of a long-term and structured follow-up, HBV/HDV co-infection did not lead to a worsening of the post-transplant clinical outcome. These data were in line with the results of Kushner et al. [9] that analyzed data from the US National Transplant Registry (UNOS), which included 152 patients with HBV/HDV co-infection and 5,435 patients with HBV monoinfection transplanted between 2002 and 2019. Although coinfected patients were younger at the time of transplants (median age 52 years vs 55 years, p < 0.001) and presented a more severe clinical picture (higher incidence of ascites and coagulopathy), post-transplant survival was not significantly different between the two groups (p = 0.77). Even in the subpopulation with hepatocellular carcinoma (HCC), survival remained comparable (p = 0.69).

Further evidence comes from Serin et al. who analyzed between 2004 and 2018, 361 transplants for HBV-related disease, of which 104 (30%) for HBV/HDV co-infection: the post-transplant survival at 82 months was 97%, viral relapse was 13.4% for HBV and 14% for HDV, while mortality was comparable between those who relapsed and those who did not (2.2 vs 7.1%; p = 0.35) [11].

Furter support comes from an Italian cohort including 1,731 liver transplant candidates between 2011 and 2020 of whom 494 (28.5%) had HDV/HBV coinfection and 1,237 (71.5%) HBV monoinfection followed until 1 February 2024 [16].

HDV/HBV coinfected patients were significantly younger, candidates for transplant mainly for decompensated cirrhosis, and had fewer cases of hepatocellular carcinoma (HCC) (26 vs 65.8%; P < 0.0001) compared with HBV monoinfected patients. HDV/HBV coinfected patients had better 5-year ITT survival (83.2%; 95% CI: 79.4–83.4%, vs 71.6%; 95% CI: 68.8–74.2%; P < 0.0001). Five years after transplant, 99.1% of HDV/HBV coinfected patients received oral nucleoside analogues, with 91.8% of patients receiving hepatitis B antigen immune globulin. HBV and HDV viral recurrences were 1.1 and 0.2%, respectively, while recurrent or de novo HCC were 8.9 and 0.3%, respectively. Therefore, also from this Italian cohort, HDV/HBV co-infected patients on the liver transplant waiting list showed more favorable outcomes than HBV mono-infected patients, both before and after transplantation [16].

Similarly, in our cohort, we did not find a statistically significant difference in overall mortality between patients transplanted for HBV monoinfection and those with HBV/HDV coinfection (15.8 vs 9.9%; p = 0.170).

The analysis of patients who developed post-transplant clinical events showed a significant association between the presence of CKD at the time of transplant and overall mortality and occurrence of clinical events, while other comorbidities such as diabetes mellitus and heart disease were not statistically associated with events. This underlines the importance of the assessment and management of renal function in the pre-transplant phase, also confirmed by the results of Kushner et al. [9].

Another key finding was the pivotal role of structured follow-up in improving outcomes. Most clinical events and deaths occurred within the first 5 years after transplantation highlighting the importance of intensive monitoring in the first years after surgery. However, the long-term stability observed beyond 10 years suggested, when appropriately managed, a lasting benefit of transplantation in HBV and HBV/HDV patients.

The present paper, however, showed several limitations. The retrospective nature of the design limited the possibility of controlling all confounding variables and introduced a potential bias in the collection of clinical data, especially with regard to intercurrent events not systematically documented in the database. The retrospective multicenter design inevitably introduced variability in data completeness, particularly for non-standardized clinical variables. The absence of systematic HDV-RNA assessment represents an important limitation, as anti HDV positivity alone does not discriminate between active infection and previous exposure. Although we emphasized the relevance of structured follow-up, we were unable to objectively assess patient adherence to follow-up protocols or correlate the frequency of visits and tests with clinical outcomes. Moreover, in our cohort, we observed a relatively high prevalence of HBV/HDV coinfection. This finding likely reflected the characteristics of the participating centers, all tertiary hepatology unit located within the same region, at high incidence of HDV infection as ultra-specialized centers, they predominantly managed patients with complex clinical profiles who required advanced multidisciplinary care from both an infectious disease and hepatology perspective. Instead, the multicenter design and the long-term follow-up (up to 20 years) represented certain strengths.

## Conclusions

In summary, our study confirmed that HBV/HDV co-infection, although associated with high clinical complexity at the transplant, did not determine a worse clinical outcome compared to HBV monoinfection. Multidisciplinary management and personalized follow-up were confirmed as key elements to improve survival and reduce complications. Early identification of high-impact comorbidities, such as CKD, represented an additional strategic objective in the management of transplant patients.

To validate and strengthen the evidence that has emerged, prospective multicenter studies will be needed to implement standardized post-transplant follow-up protocols and evaluate the actual adherence of patients to clinical-virological surveillance programs. Considering the burden of renal failure highlighted in our study, risk stratification based on comorbidities such as chronic renal failure should guide prevention and early management strategies in transplant candidates and transplant recipients.

## Supplementary Information

Below is the link to the electronic supplementary material.

Supplementary file1 (DOCX 20 KB)

## Data Availability

The data presented in this study are available on request from the corresponding author.
