# Supplementary material for: Impact of HDV infection on post-transplant outcomes in patients transplanted for HBV-related liver disease: results from a multicenter cohort study in Southern Italy
Source: Infection. 2025 Dec 15;54(2):771–80. doi: 10.1007/s15010-025-02707-5 (PMC13021802; doi:10.1007/s15010-025-02707-5)
Supplement: Supplementary file 1 — Supplementary file1 (DOCX 20 KB) [file 15010_2025_2707_MOESM1_ESM.docx]

**Supplementary table 1**: demographic and clinical characteristics of the enrolled patients

|  | Total HBV pts |
| --- | --- |
| Total patients observed | 257 (100%) |
| Gender M n° (%) | 194 (75,5%) |
| Age at transplant  Median and range | 55 (49;61) |
| No° (%)* patients with   - IVDA - Ethyl abuse - Familiarity with HbsAg | 21(8,3%)  38 (15,1%)  76 (35,7%) |
| Years of follow-up, Median (IQR) | 7 (4;12) |
| No° (%) transplant patients   - <2010 - 2010-2015 - 2016-2020 - >2020 | 65 (25,4%)  61 (23,8%)  89 (34,8%)  41 (16,0%) |
| Transplanted for  - Cirrhosis n (%)  - HCC n (%) | 201 (98,0%)  115 (59,9%) |
| No° (%)/ tested* transplant patients   - with NUC - HBV DNA neg - HDV RNA pos - Anti-HCV - Anti-HIV | 178/178 (100%)  103/132 (78%)  17/33 (51,5%)  26/252 (10,3%)  0/188 (0%) |
| No° (%)/ tested* transplant patients   - With diabetes mellitus - CKD - Heart disease - Psychiatric pathology | 41/183 (22,4%)  16/182 (8,8%)  25/188 (13,3%)  15/176 (8,5%) |
| N° (%)patients with at least one clinical event (hepatic or extra-hepatic) | 84 (32,7%) |
| N° (%)patients with at least one clinical event   - In the first 5 years after transplant - From the 6th to the 10th year post transplant - After the 10th year post transplant | 77 (92,8%)  5 (6,0%)  1 (1,2%) |
| N° (%)patients with at least one hepatic event (cirrhosis, HCC, HBV relapse, decompensation) | 19 (7,4%) |
| N° (%)patients with at least one hepatic event   - In the first 5 years after transplant - From the 6th to the 10th year post transplant - After the 10th year post transplant | 17 (89,5%)  2 (10,5%)  0 (0%) |
| N° (%)patients with at least one extra-hepatic event (renal failure, new onset neoplasm) | 72 (28,0%) |
| N° (%) patients with at least one extra-hepatic event   - In the first 5 years after transplant - From the 6th to the 10th year post transplant - After the 10th year post transplant | 67 (93,1%)  4 (5,6%)  1 (1,4%) |
| N° (%) patients with   - HBV relapse - HCC - cirrhosis - hepatic decompensation - renal failure - new onset cancer | 0 (0,0%)  13 (5%)  6 (2,3%)  2 (0,7%)  57 (22,1%)  32 (12,4%) |
| N° (%) patients with   - death related to hepatic event - death not related to hepatic event - overall deaths | 9 (3,5%)  22 (8,6%)  31 (12,1%) |
| N° (%)patients with liver-related death   - In the first 5 years after transplant - From the 6th to the 10th year post transplant - After the 10th year post transplant | 7 (77,8%)  2 (22,2%)  0 (0%) |
| N° (%)patients with death not related to hepatic events   - In the first 5 years after transplant - From the 6th to the 10th year post transplant - After the 10th year post transplant | 13 (59,1%)  7 (31,8%)  2 (9,1%) |
| N° (%)patients with overall death   - In the first 5 years after transplant - From the 6th to the 10th year post transplant - After the 10th year post transplant | 21 (67,7%)  8 (25,8%)  2 (6,5%) |

**Supplementary table 2:** Number of patients included in the study in each center.

| **Center** | **N° of patients** |
| --- | --- |
| **University of Campania “Luigi Vanvitelli”** | 51 |
| **University of Naples “Federico II”** | 22 |
| **University of Salerno** | 11 |
| **AORN Cardarelli** | 85 |
| **AORN Sant’Anna e San Sebastiano** | 36 |
| **Gragnano Hospital** | 52 |
